# The Influence of Social Network Characteristics on Peer Clustering in Smoking: A Two-Wave Panel Study of 19- and 23-Year-Old Swedes

**DOI:** 10.1371/journal.pone.0164611

**Published:** 2016-10-11

**Authors:** Alexander Miething, Mikael Rostila, Christofer Edling, Jens Rydgren

**Affiliations:** 1 Department of Sociology, Stockholm University, SE-106 91 Stockholm, Sweden; 2 Centre for Health Equity Studies (CHESS), Stockholm University/Karolinska Institutet, SE-106 91 Stockholm, Sweden; 3 Department of Sociology, Lund University, SE-221 00 Lund, Sweden; Nanyang Technological University, SINGAPORE

## Abstract

**Objectives:**

The present study examines how the composition of social networks and perceived relationship content influence peer clustering in smoking, and how the association changes during the transition from late adolescence to early adulthood.

**Methods:**

The analysis was based on a Swedish two-wave survey sample comprising ego-centric network data. Respondents were 19 years old in the initial wave, and 23 when the follow-up sample was conducted. 17,227 ego-alter dyads were included in the analyses, which corresponds to an average response rate of 48.7 percent. Random effects logistic regression models were performed to calculate gender-specific average marginal effects of social network characteristics on smoking.

**Results:**

The association of egos’ and alters’ smoking behavior was confirmed and found to be stronger when correlated in the female sample. For females, the associations decreased between age 19 and 23. Interactions between network characteristics and peer clustering in smoking showed that intense social interactions with smokers increase egos’ smoking probability. The influence of network structures on peer clustering in smoking decreased during the transition from late adolescence to early adulthood.

**Conclusions:**

The study confirmed peer clustering in smoking and revealed that females’ smoking behavior in particular is determined by social interactions. Female smokers’ propensity to interact with other smokers was found to be associated with the quality of peer relationships, frequent social interactions, and network density. The influence of social networks on peer clustering in smoking decreased during the transition from late adolescence to early adulthood.

## Introduction

The strong association between the initiation of smoking and peer-group smoking during adolescence has prompted numerous studies to examine the possible causal links between individuals’ and peers’ smoking behavior [1–4]. A particular research focus has been to understand the development of smoking during the transition from adolescence to early adulthood [5]. Research on adolescents’ smoking behavior is relevant because adolescence has been identified as the most critical period for the initiation of smoking [6]. Those who start smoking in adolescence have an increased probability of becoming life-long smokers, drastically increasing their risk of cardiovascular and respiratory diseases, cancer, and premature mortality [7]. Taking up smoking a few years later significantly decreases the propensity for life-long smoking [8,9]. Smoking prevention during adolescence, therefore, is an effective public health strategy.

Adolescents’ smoking initiation coincides with increased peer interaction, suggesting that social ties play a salient role in the onset and continuation of smoking [10,11]. During adolescence, individuals are particularly receptive to the influences of peer groups and social networks that facilitate the spread of certain group norms and attitudes, but also of health behaviors like smoking [12]. Smoking commonly begins in the presence of friends [2,13]. Smoking in this context signals conformity, expresses group cohesion, and reflects the social codes and norms present in the peer group [12,14,15]. Behavior contrary to these norms may violate group conformity and even result in group expulsion [16]. The ego-alter association in smoking and other health behaviors depend on how social relationships are structured. Social network characteristics, the degree of group cohesion, and the strength of relationships facilitate the transmission of similar social behaviors [16]. For example, more frequent social interactions were found to increase the probability of smoking [17]. Previous research has further demonstrated that not only structural characteristics but also the quality of social ties determines ego-alter homogeneity in various health behaviors [18,19]. Specifically, trusting social relationships were shown to reveal ambivalent associations with health outcomes and health behaviors: Otherwise regarded as beneficial for health and well-being, social ties of high quality were also found to facilitate the co-evolution of smoking [14]. Positive associations between relationship quality and smoking may additionally reflect that smokers are more popular than non-smokers [20].

Moreover, earlier research has indicated notable gender differences in social interactions, particularly regarding the intensity with which males and females engage in social relationships. Adolescent females were found to be more frequently involved in intimate and reciprocal social relationship than males [21–23]. If smoking behavior is conditional on the strength and intensity of social interaction, these results imply that young women may be more prone to take up smoking.

The association between peer clustering in smoking and other health behaviors is likely to be a consequence of two simultaneously occurring mechanisms: *induction* and *homophily*. The induction hypothesis—also termed social contagion—suggests that peer groups influence the ego and presumes a certain degree of susceptibility to adopting behaviors, norms, and attitudes defined by the peer group. By contrast, the homophily hypothesis acknowledges that individuals select their peers according to certain characteristics represented in the peer group. There is an enduring debate in the social network literature on the predominant role of either induction or homophily [24]. However, there is increasing agreement that both mechanisms are mutually compatible, as autocorrelation occurs between ego’s and alters’ behaviors [25–27]. To unambiguously disentangle the two mechanisms would require data on the reciprocity of social relations between individuals and their peers over time [15,24]. Because the use of two-wave panel data in the present study makes resolving the puzzle impossible, a specific focus will be on the transition from adolescence to adulthood with the aim of identifying implications regarding the causal direction of associations. As previous research has proposed, peer influences diminish when young people grow into adulthood, while selection mechanisms become increasingly important [28–30]. Relationship duration as it relates to peer clustering in smoking may therefore provide an avenue for exploring whether ego-alter correlation in smoking grows or diminishes over time. Whereas long-lasting social relationships, for example, are believed to indicate selection [31], new network members, by contrast, are assumed to adopt more easily the predominant social behaviors in peer networks [32].

The spread of social behaviors may depend on the composition of the social network. Smoking tends to spread more easily the more similar egos and alters are, which is reflected in the degree of group cohesion [15]. Previous research has suggested that network density, that is, the number of social ties within peer networks, may facilitate behavioral similarity [33–35]. Empirical evidence on the development of peer clustering in smoking during the transition period from adolescence to adulthood is relatively scarce. Studying network dynamics and changes over time may therefore contribute to our understanding of the interdependence of egos’ and alters’ smoking and its underlying mechanisms. In particular, the transition period from adolescence to early adulthood constitutes a compelling time frame for analyzing young individuals’ smoking in relation to peers’ smoking, because social network formations and the susceptibility to peer influences are likely to vary at younger ages. Increasing peer interactions in late adolescence may also counter the preventive effect of parental monitoring on smoking behavior that was demonstrated for younger adolescents [20]. Given the different smoking patterns by boys and girls and the distinct way they interact with peers, it is advisable to conduct gender-specific analyses.

### Aims of the study

The aim of this study is to explore the influence of network composition and relationship aspects (i.e., how often egos meet alters, friendship network trust and relationship quality, network density, and relationship duration) on the ego-alter relationship in smoking. Furthermore, the study explores the extent to which the associations change during the transition from late adolescence to early adulthood.

The study is guided by the following research questions:

1. How does the association between ego’s and alters’ smoking develop during the transition from late adolescence to early adulthood?
2. How do trust in peers, relationship quality with peers, and network composition influence the relation between ego’s and alters’ smoking during the transition from late adolescence to early adulthood?
3. Do gender-specific social interactions influence the ego-alter relationship in smoking?

Using two-wave panel data with ego-alter dyads, the study investigates the main association with ego’s smoking and, in a second step, explores the three-way interactions between peer clustering in smoking, relationship characteristics, and the transition to adulthood. Gender-specific analyses will be performed as smoking pattern, social relations, and network characteristics are assumed to differ between males and females.

## Data and Methods

The present study analyzes data derived from the first and second wave of the Swedish survey “*Social Capital and Labor Market Integration”*. Respondents’ information in this study was anonymized and de-identified prior to analysis. The Ethical Review Board of Stockholm approved the study (2008/580-31). The data contain information on the everyday life and social networks of young Swedes born in 1990. Most respondents had reached the age of 19 when the initial wave was collected in 2009, and were 23 years old in the follow-up in 2013. The strategic sample targeted 5,695 individuals, of which almost two-thirds had a foreign background with at least one parent born either in Iran or former Yugoslavia. The data were collected by a telephone survey conducted by Statistics Sweden. Due to the relatively widespread use of unregistered prepaid phones in this age group, phone numbers were unavailable or could not be retrieved, which is the primary reason for non-responses in the study sample. In total, 2,942 interviews were successfully completed. The corresponding response rate was 51.7%. The follow-up study targeted the initial sample from the first wave and resulted in a response rate of 39.4%. The present study comprised 2,774 individuals, which corresponds to 17,227 ego-alter dyads with full information on all study variables and an overall average response rate of 48.7%. Of those, 6,248 dyads were drawn from the first panel wave, 10,024 dyads from both waves, and 955 dyads were from the second wave only.

The interviews involved questions about the respondents’ everyday lives, health behavior, and social networks. Respondents (“ego”) were asked to name up to five people (“alter”) with whom they have rather close relationships and interact most frequently. Nominated alters could be friends but could also include school and work mates, relatives, siblings, and romantic partners.

### Outcome variable

Ego’s smoking, which was the outcome variable, was based on the question “Do you smoke daily?” Daily smokers were categorized as *smoker* or occasional smokers; nonsmokers were classified as *nonsmoker*.

### Network composition variables

Frequency of meeting was based on asking how often ego and alter meet. The responses ranged along six categories from “daily” to “rarely/never.” How long ego and alter had known each other was assessed with the question “How many years have you known each other?” If they knew each other less than one year, respondents were prompted to provide the duration in months. For relationships longer than one year, they were asked to give the number of years. Relationship durations of less than one year were coded as 0, all other categories denote the duration in years. Network density assesses the share of alters interconnected with each other within each network and depicts whether egos’ networks are more homogenous or heterogeneous. The information was derived from egos’ responses to the question about whether respective alters knew each other. Value “0” denotes that alters did not know other at all; “0.5” that half of all alters knew each other, and “1” that all alters knew each other within the network.

### Relationship content

Alters’ smoking behavior was derived from the egos’ responses on the question whether alter # smokes, and it was then dichotomized into *smoking* and *non-smoking*. Relationship content was based on the egos’ ratings of self-perceived *relationship quality* and *trust in alters*. *Relationship quality* was based on the question, “How is good is your relationship with alter #?” and included five response options ranging from 1 (not at all good) to 5 (very good). *Trust in alters* was based on the question, “How much do you rely on alter #?” with corresponding response options ranging from 1 (not at all) to 5 (very much). Due to few individuals with very low ratings in *trust* and *relationship quality*, the two lowest categories of both variables were merged into a single one.

### Individual control variables

Because all respondents were born in 1990, the *age* variable shows the respondents’ age at the time they were interviewed. Accordingly, it also reflects whether data were derived from the first or second wave of the survey sample. Nearly half of the respondents in the study sample have at least one parent who came from either Iran or former Yugoslavia. The migration background variable therefore distinguishes among Swedish, Iranian and Yugoslavian descent. Employment status reflects ego’s socioeconomic situation and indicates whether the ego studies, works, is unemployed, or does something else other than the aforementioned activities. Civil status indicates whether the ego is or is not in a relationship or married. Respondents’ highest educational attainment was used as a control for the socioeconomic position. Educational attainment consists of three categories comprising primary, secondary and post-secondary levels.

### Modeling strategy

The analysis was performed with random effects logistic models that accounted for the two-wave panel structure of the data. Results are reported as odds ratios along with average marginal effects (AME). The latter show the discrete changes in the probability of egos’ smoking in relation to social network characteristics. Analyses were based on multiple observations per individual: egos were clustered in up to five ego-alter dyads per panel wave, which resulted in up to ten observations for each ego. As a result, the analysis also accounted for the number of nominated alters. The obtained marginal effects consequently reflect the associations at the dyad-level and show how each social tie contributes to the ego’s smoking probability. Ego’s probability of smoking may accumulate with the influences of multiple social ties. Individual level inferences, however, depend on the degree of interdependence between other network members. The increase of effects from additional alters in a network is not linear because additional alters have a rather diminishing effect on ego. Disregarding the interdependence between alters could result in an overestimation of effects at the ego level [36]. In order to correct for the deflation of standard errors and widened confidence intervals imposed by the dyad-level analysis, a cluster robust function was used to obtain individual specific confidence intervals. The modeling procedure involved the estimation of main effects on ego’s smoking probability and the calculation of interaction effects between the co-evolution of smoking, network characteristics, and egos’ age. The three-way interaction term and its implementation in the random effects logistic regression model is specified in the following equation:

$$\text{Pr}{\left(\text{E}\text{GO SMOKES}=1\right)}_{it}=\beta_{0}+\beta_{1}\text{A}{\text{GE}}_{it}+\beta_{2}{\text{A}\text{LTER SMOKES}}_{it}+\beta_{3}{\text{N}\text{ETWORK VARIABLE}}_{k,it}+\beta_{4}{\text{A}\text{GE}}_{it}*{\text{A}\text{LTER SMOKES}}_{it}*{\text{N}\text{ETWORK VARIABLE}}_{k,it}+\ldots +\beta_{m}{\text{X}}_{k,it}+u_{i}+\varepsilon_{it}$$

The term X_*k*,*it*_ denotes the control variables included in the model, and *β*_*m*_ represents the corresponding coefficients. Odds ratios with respective confidence intervals were calculated to demonstrate whether interaction terms (with and without age variable) were significant. In order to provide a more detailed outline of how alters’ smoking relates to egos’ smoking at specific ages and values of the network variables, interactions based on average marginal effects were calculated and plotted. Despite nonsignificant interaction effects indicated by odds ratios, the corresponding interactions (i.e., cross-differences between variables) in the average marginal effect metric may be significant (and vice versa) [37,38]. Because marginal effects capture better how the effect of one variable changes when another variable changes, the interpretation of findings was based on this metric only.

## Results

Table 1 provides the variable distributions and illustrates the demographic composition of the study sample. The figures suggest that more females than males are daily smokers. The individual level variables show that most of the respondents are of Swedish origin. A majority of respondents study or work. Nearly two-thirds in the sample are singles. Most of the egos have at least a secondary school degree. Because a large share of respondents is still in education, the proportion of post-secondary educational attainment is relatively low. The variation in smoking behavior during the observation period is rather low, but nevertheless indicates a relative increase in smoking for males and a decrease for females. Whereas the proportion of male smokers remains stable, the share of female smokers notably decreases from age 19 to age 23.

**Table 1:** Distribution of social network variables and egos' characteristics at the dyad-level.

Variables	Males		Females		Total	
	Dyads	%	Dyads	%	Dyads	%
Ego's age						
* 19 years*	5,726	65.2	5,534	65.5	11,260	65.4
* 23 years*	3,056	34.8	2,911	34.5	5,967	34.6
Ego smokes daily						
* Yes*	1,172	13.4	1,551	18.4	2,723	15.8
* No*	7,610	86.7	6,894	81.6	14,504	84.2
Ego's smoking transition						
* Smoker at age 19*	766	13.4	1,151	20.8	1,917	17.0
* Smoker at age 23*	406	13.3	400	13.7	806	13.5
* Nonsmoker at age 19 → Smoker at age 23*	150	1.7	113	1.3	263	1.5
* Smoker at age 19 → Nonsmoker at age 23*	111	1.3	177	2.1	288	1.7
**Network variables**						
Alter smokes						
* Yes*	2,550	29.0	2,600	30.8	5,150	29.9
* No*	6,232	71.0	5,845	69.2	12,077	70.1
How often ego/alter meet each other						
* Daily*	1,577	18.0	1,624	19.2	3,201	18.6
* Several times a week*	2,822	32.1	2,342	27.7	5,164	30.0
* Once a week*	2,294	26.1	1,960	23.2	4,254	24.7
* Once a month*	1,620	18.5	1,892	22.4	3,512	20.4
* Few times a year*	375	4.3	548	6.5	923	5.4
* Seldom or never*	94	1.1	79	0.9	173	1.0
Quality of relationship with alter						
* (1) Not good at all*	128	1.5	118	1.4	246	1.4
* (2) …*	1,070	12.2	1,153	13.7	2,223	12.9
* (3) …*	3,124	35.6	2,740	32.5	5,864	34.0
* (4) Very good*	4,460	50.8	4,434	52.5	8,894	51.6
Trust to alter						
* (1) Not at all*	282	3.2	236	2.8	518	3.0
* (2) …*	1,074	12.2	947	11.2	2,021	11.7
* (3) …*	2,525	28.8	2,162	25.6	4,687	27.2
* (4) Very much*	4,901	55.8	5,100	60.4	10,001	58.1
Relationship duration	*Mean*:	*SD*:	*Mean*:	*SD*:	*Mean*:	*SD*:
* Time in years*	7.51	5.65	7.01	6.00	7.26	5.83
Network density	*Mean*:	*SD*:	*Mean*:	*SD*:	*Mean*:	*SD*:
* Low (0) to high (1)*	0.78	0.26	0.72	0.26	0.75	0.26
**Individual variables**						
Ego's parents’ country of birth						
* Sweden*	4,338	49.4	4,344	51.4	8,682	50.4
* Iran*	1,818	20.7	1,817	21.5	3,635	21.1
* Former Yugoslavia*	2,626	29.9	2,284	27.1	4,910	28.5
Ego's civil status						
* Single*	5,750	65.5	4,849	57.4	10,599	61.5
* With partner*	2,954	33.6	3,457	40.9	6,411	37.2
* Married*	78	0.9	139	1.7	217	1.3
Ego's employment status						
* Study only*	2,922	33.3	2,606	30.9	5,528	32.1
* Work only*	2,884	32.8	2,942	34.8	5,826	33.8
* Work and study*	1,029	11.7	1,669	19.8	2,698	15.7
* Unemployed*	557	6.3	191	2.3	748	4.3
* Do something else*	1,390	15.8	1,037	12.3	2,427	14.1
Ego's educational attainment						
* Primary school or no degree*	483	5.5	383	4.5	866	5.0
* Secondary school degree*	7,789	88.7	7,308	86.5	15,097	87.6
* Post-secondary/ academic degree*	510	5.8	754	8.9	1,264	7.3
**Total of ego-alter dyads**	**8,782**	**100.0**	**8,445**	**100.0**	**17,227**	**100.0**

Table 2 shows the gender-specific associations between social network characteristics and egos’ daily smoking. In order to present a consistent metric for main and interaction effects, results are displayed as odds ratios and AME’s. The probability metric based on AME’s allows a more straightforward interpretation of the regression model and is therefore used for the description of results. Egos’ age was included to account for the changes in the probability of smoking between ages 19 and 23. Among males, age is not associated with smoking behavior. By far the strongest predictor of smoking is peer smoking: males who named a smoking alter have a 7 percent increase in the probability of smoking compared to those who named a nonsmoker. The corresponding odds ratio (OR = 8.12, 95% CI 5.58 to 11.81) denotes that males’ odds of smoking are 8 times higher for those who have a smoking peer in the personal network compared to those who interact with nonsmokers only. The associations in the female sample are even stronger: a smoking peer increases females’ probability of smoking by 17.6 percent. Table 2 also shows how aspects of social networks relate to egos’ smoking behavior. For example, a male who meets alter once a week rather than every day reduces his smoking probability by 2 percent compared to males who interact with alters on a daily basis. For females the respective decrease is nearly 5 percent. Friendship duration measured in years indicates a nonsignificant negative association with males’ and females’ smoking. Network density did not reveal significant main effects on males’ or females’ smoking behavior.

**Table 2:** Random effects logistic regression: Odds ratios and average marginal effects for daily smoking by gender.

Variables	Males				Females			
	OR	95% CI	AME	95% CI	OR	95% CI	AME	95% CI
Ego: age 23	**1.29**	(0.79; 2.10)	**0.009**	(-0.008; 0.027)	**0.75**	(0.48; 1.17)	**-0.021**	(-0.054; 0.011)
Alter smokes	8.12**	(5.58; 11.81)	0.074**	(0.044; 0.104)	11.16**	(7.66; 16.28)	0.176**	(0.145; 0.207)
How often ego/alter meet each other								
* Daily*	**Ref**.		**Ref**.		**Ref**.		**Ref**.	
* Several times a week*	0.68*	(0.48; 0.96)	**-0.016**†	(-0.032; 0.000)	**0.91**	(0.66; 1.25)	**-0.008**	(-0.036; 0.020)
* Once a week*	0.60*	(0.39; 0.91)	-0.020*	(-0.038; -0.002)	0.52**	(0.36; 0.75)	-0.049**	(-0.077; -0.021)
* Once a month*	0.57*	(0.35; 0.92)	-0.021*	(-0.041; -0.002)	0.50**	(0.34; 0.75)	-0.051**	(-0.081; -0.022)
* Few times a year*	**0.62**	(0.29; 1.33)	**-0.019**	(-0.046; 0.008)	0.38**	(0.21; 0.71)	-0.067**	(-0.103; -0.030)
* Seldom or never*	**0.40**	(0.12; 1.32)	**-0.031**†	(-0.062; 0.001)	0.17**	(0.05; 0.57)	-0.100**	(-0.144; -0.056)
Quality of relationship with alter								
* (1) Not good at all*	**Ref**.		**Ref**.		**Ref**.		**Ref**.	
* (2) …*	**1.92**	(0.69; 5.30)	**0.014**	(-0.005; 0.033)	**1.24**	(0.52; 2.92)	**0.014**	(-0.040; 0.069)
* (3) …*	**2.37**	(0.79; 7.13)	0.021*	(0.000; 0.041)	**1.19**	(0.48; 2.96)	**0.012**	(-0.046; 0.069)
* (4) Very good*	**2.98**†	(0.98; 9.10)	0.029*	(0.006; 0.052)	**1.42**	(0.55; 3.71)	**0.025**	(-0.037; 0.086)
Trust to alter								
* (1) Not at all*	**Ref**.		**Ref**.		**Ref**.		**Ref**.	
* (2) …*	0.44*	(0.20; 0.93)	**-0.038**†	(-0.082; 0.006)	**0.66**	(0.35; 1.25)	**-0.035**	(-0.092; 0.023)
* (3) …*	0.45*	(0.21; 0.97)	**-0.037**	(-0.082; 0.008)	**0.59**	(0.31; 1.15)	**-0.042**	(-0.102; 0.017)
* (4) Very much*	**0.47**†	(0.21; 1.05)	**-0.035**	(-0.081; 0.011)	**0.61**	(0.30; 1.25)	**-0.04**	(-0.104; 0.024)
Relationship duration								
* Between 0 and 19 years*	**0.98**†	(0.95; 1.00)	**-0.001**	(-0.002; 0.000)	**0.99**	(0.97; 1.01)	**-0.001**	(-0.002; 0.001)
Network density								
* Continuous scale from 0 (low density) to*	**2.07**	(0.83; 5.12)	**0.026**	(-0.007; 0.059)	**0.85**	(0.41; 1.74)	**-0.012**	(-0.065; 0.041)
* 1 (high density)*								
Two-way interactions								
* Alter smokes × Age*	0.56*	(0.34; 0.91)			0.32**	(0.19; 0.54)		
* Alter smokes × How often ego/alter meet*	0.70**	(0.56; 0.88)			0.81*	(0.68; 0.97)		
* Alter smokes × Quality of relationship*	**1.29**	(0.95; 1.77)			1.37*	(1.05; 1.78)		
* Alter smokes × Trust to alter*	**1.10**	(0.85; 1.43)			**1.24**†	(0.97; 1.59)		
* Alter smokes × Relationship duration*	**0.99**	(0.94; 1.03)			**0.98**	(0.95; 1.01)		
* Alter smokes × Network density*	**1.32**	(0.51; 3.41)			4.33**	(1.82; 10.33)		
Three-way interactions								
* Alter smokes × Age × How often ego/alter meet*	**0.94**	(0.63; 1.40)			**1.05**	(0.76; 1.45)		
* Alter smokes × Age × Quality of relationship*	**0.78**	(0.40; 1.50)			**0.75**	(0.40; 1.39)		
* Alter smokes × Age × Trust to alter*	**1.09**	(0.64; 1.86)			**1.03**	(0.59; 1.78)		
* Alter smokes × Age × Relationship duration*	**1.06**	(0.98; 1.15)			**0.99**	(0.92; 1.07)		
* Alter smokes × Age × Network density*	**0.36**	(0.05; 2.90)			**0.93**	(0.12; 7.55)		
No. of dyads	8,782		8,782		8,445		8,445	
No. of individuals	1,418		1,418		1,356		1,356	

^†^ *p* < 0.10;

* *p* < 0.05;

** *p* < 0.01

All variables mutually adjusted, including migration background, employment status, civil status and educational attainment (coefficients not shown)

Random effects parameters not shown

The associations between smoking and self-perceived social support are somewhat stronger, but are significant only in the male sample. Very good relationships with peers increase young men’s probability of smoking by nearly 3 percent compared to poor relationship quality. In the female sample, relationship quality does not reveal significant associations with smoking. Very low ratings in *trust in alters* reveal elevated smoking probabilities among males only. A similar pattern is shown for females but estimates remain nonsignificant. In contrast to the gradual smoking pattern in *relationship quality*, the smoking propensity does not vary by medium or high levels of trust.

### Interaction analyses

The main intent of this study was to explore the extent to which the associations between egos’ and alters’ smoking varies by influences from third variables. Significant interactions then imply that the strength of peer clustering in smoking depends on other network aspects. Age-specific plots account for the distinct associations in late adolescence and early adulthood.

**Fig 1:**
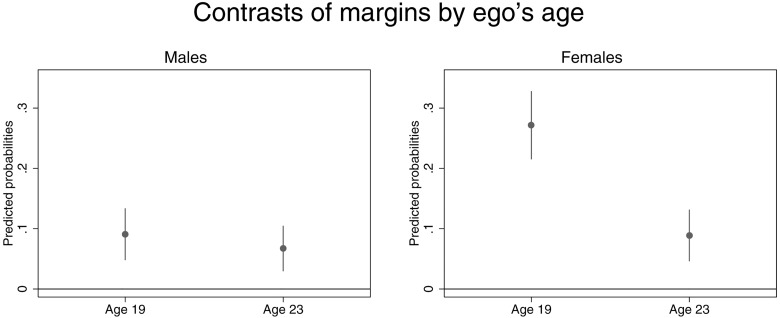
Gender-specific interactions of ego’s age with ego-alter relationship in smoking: Predicted probabilities based on contrasts of average marginal effects with 95% confidence intervals. Coefficient markers show the discrete change of egos’ smoking probability when alters smoke relative to their probability when alters are nonsmokers (reference y = 0).

**Fig 2:**
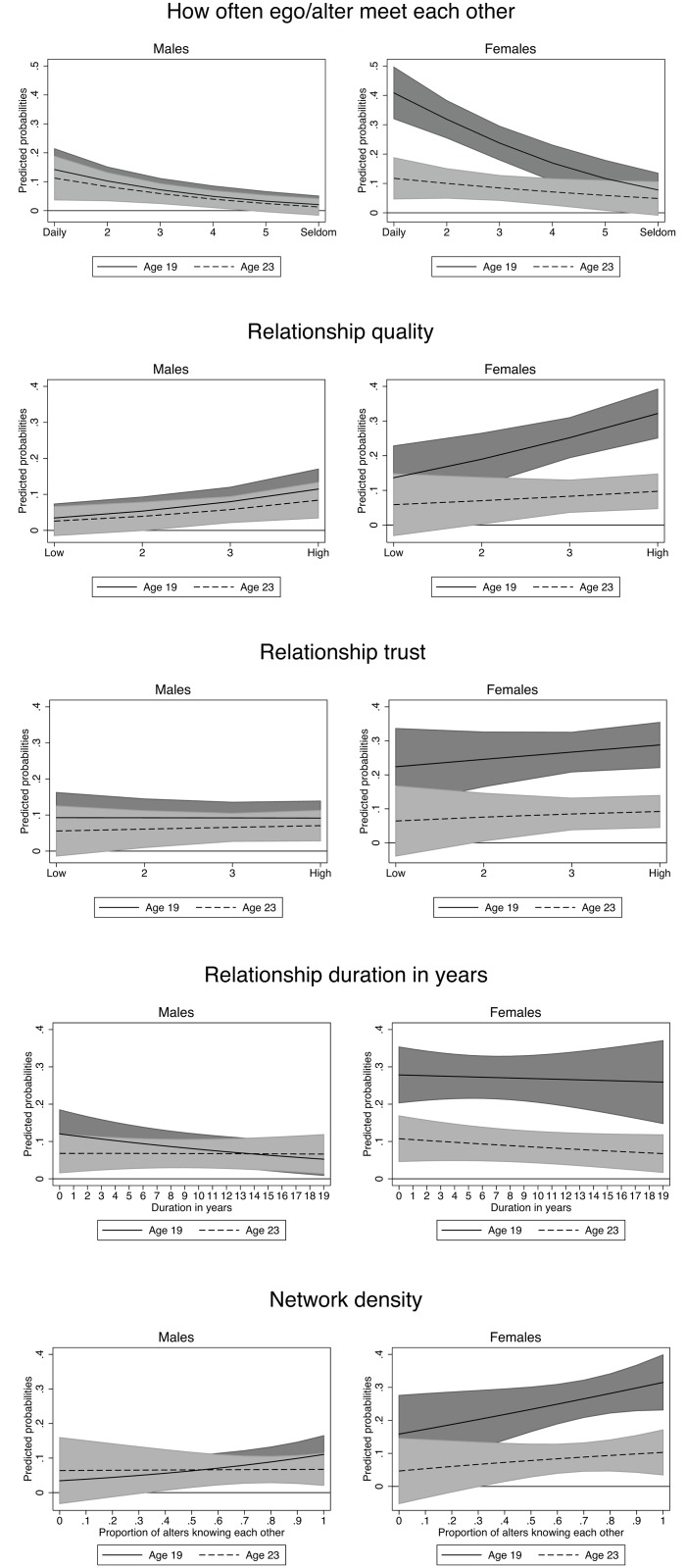
Gender-specific interactions between social network characteristics and ego-alter relationship in smoking by age: Predicted probabilities based on contrasts of average marginal effects with 95% confidence intervals. Coefficient lines show the discrete change of egos’ smoking probability when alters smoke relative to their probability when alters are nonsmokers (reference y = 0). All network covariates were implemented as continuous variables.

The interaction plots in Figs 1 and 2 depict the *contrast of margins*, that is, the discrete change between having smoking and non-smoking peers. The coefficient markers describe the increase in smoking probability of egos with contact with smoking alters compared to egos who socialize with nonsmokers. The interaction with age in Fig 1 shows that the ego-alter association in females’ smoking significantly decreases from age 19 to 23: When interacting with a smoking peer, the dyadic probability of smoking for a 23-year-old female is 17 percent lower than for a 19-year-old female. In the male sample, ego’s age was not related to peer clustering in smoking. With regard to the network characteristics, the results indicate that females’ peer relationships generally impose stronger influences on the association between own and others’ smoking behavior. The calculations based on *How often ego and alter meet each other* demonstrate that ego’s and alters’ smoking behavior becomes increasingly similar the more often they meet. This increase is stronger for females. Perceived emotional support based on *relationship quality* revealed a positive gradient in smoking probability at age 19 that was markedly stronger for females. A gradient at age 23 was still notable but nonsignificant as the overlapping confidence intervals in lower and upper range of *relationship quality* demonstrate. The corresponding analysis with *trust* showed a weak positive but nonsignificant association in females only.

Fig 2 shows the interactions with friendship duration and the degree of network heterogeneity. Relationship duration measured in years reveals a weakly negative association with peer clustering in smoking but nevertheless suggests that egos’ smoking propensity gradually decreases the longer ego and alter have known each other. The age-specific interaction plots indicate that friendship duration is more influential at age 19. The interaction with *network density* shows to what extent the smoking patterns in ego-alter dyads vary by social network diversity. Higher values on the x-axis refer to a higher share of peers who know each other, and thus indicate a lower degree of network diversity. The slopes, especially the one for females, demonstrate that the ego-alter homogeneity in smoking is stronger the more alters know each other within the same network.

## Discussion

The study aimed to investigate the similarity in ego’s and alters’ smoking behavior and how the associations are influenced by additional social network characteristics. A two-wave panel design was used to explore how associations changed during the transition from late adolescence into early adulthood. Consistent with previous research, the findings confirm the strong association between individual and peer smoking. Smoking by peers remained the strongest predictor of ego’s smoking, even after controlling for individual factors and network characteristics. Stronger associations with peer smoking among females indicate that females interact differently with peers than males, and probably engage in more intense relationships with a higher degree of intimacy and reciprocity [21,39]. Men’s smoking behavior, by contrast, appears to be more independent of peer interactions.

The most salient findings of this study are the decreased associations between smoking and network influences from ages 19 to 23 which may reflect reduced susceptibility to peer group pressure resulting from increased autonomy and more stable personal traits in early adulthood compared to late adolescence [29]. The female sample in particular revealed that peer network influences on smoking behavior diminish when entering early adulthood. This gender pattern in smoking cessation was observed in earlier Swedish and Finnish studies [40,41]. The stronger network influences in late adolescence shown in the present study may reflect the fact that 19-year-olds have fewer options for engaging in heterogeneous networks. At age 19, school still represents the primary setting for peer interactions. After age 19, when people have generally started higher education or work, the chances for diversifying social contacts, thereby diffusing possible network influences, increase [42]. Previous research has also shown that attitudes toward smoking and nonsmoking become clearer with increasing age [43]. The lowered smoking probability of 23-year-old women may mirror gender-specific social norms and lifestyles. Swedish women have previously been shown to engage more often in healthful activities than men [44]. Estimates involving *trust* and *relationship quality* showed remarkably different patterns on egos’ smoking propensity: associations between *relationship quality* and smoking were found to obscure the otherwise beneficial effects of constructive social relations on health and well-being. Smoking probability gradually increased with higher ratings on relationship quality, probably because smoking egos were influenced by smoking alters and non-smoking egos by non-smoking alters. The results indicate the ambivalence of constructive social relationships. Stronger social cohesion may enforce group norms that either enhance or lessen the spread of smoking behaviors [12].

The increased smoking probability induced by the lack of *trust in peers* somewhat counters the findings for *relationship quality* and suggests that both variables measure different relationship aspects. Smokers seem to mind about their co-smokers but do not necessarily perceive them as trustworthy. The negative association with trust is in line with previous research: smoking along with an absence of trust may reflect a stress reaction due to a lack of social support in destructive social relations [45]. The interaction analysis with *trust* and *relationship quality* confirms the results of the main effects: Whereas *trust* revealed hardly detectable influences on peer clustering in smoking, *relationship quality* was more clearly associated. In particular, the smoking behavior of 19-year-old females was found to be more similar to that of smoking peers when social relations are perceived as good. Stronger gradients at age 19, for both males and females, suggest that *relationship quality* is more influential in late adolescence. At that age, self-esteem, impulse control, and willpower may not be as strongly developed as in adulthood [46], implying that adolescents are less able to resist to group dynamics transmitted through well-functioning social relations [4,18]. In addition, the positive correlation between the *frequency of meetings* and smoking behavior underlines that the intensity of social interactions and real life contacts to peers matters. It is further possible that smokers create more occasions to meet with smoking peers, which would suggest endogenous correlations between smoking and some of the measures in this study. For example, smokers’ higher relationship quality as well as the correlations with *trust* could be a cause or consequence of smoking. When considering smoking as a precursor of relationship quality, co-smokers may develop stronger social ties compared to non-smoking dyads, which increases smokers’ perceptions of relationship quality.

The findings based on friendship duration indicate that long-term peer relations rather weaken the ego-alter relationship in smoking. Long-lasting peer relationships reduce the ego’s smoking propensity, but they also imply that relationships with nonsmoking peers tend to be more persistent and stable. One may therefore conclude that nonsmoking peers in long-lasting social relationships play a modeling role in influencing egos’ behaviors [47] and thus function as social control with a preventive effect on smoking [48]. Accordingly, the short-term peer relationships of smokers with other smokers may reflect that smokers easily enter relationships and fraternize more impulsively with other smokers. For example, the larger contact surface of “social smokers” appears to facilitate more opportunities to gather with other smokers [49].

Network heterogeneity was nonsignificant for egos’ smoking but revealed a gradient on peer clustering in smoking: Whereas smokers tend to engage in rather homogenous network formations with other smokers, nonsmokers tend to gather in multiple peer settings. The greater constraint of heterogeneous network formations on smoking behavior has been previously recognized: individuals who interact in several contexts smoke less because smoking may cause disapproval by nonsmoking peers [50,51]. In addition, group cohesion and peer-group pressure are likely to vary, depending on the degree of network heterogeneity: networks with a high proportion of interconnected peers may facilitate a high degree of group cohesion and raise potential peer group pressure [16,52,53]. Correspondingly, in heterogeneous peer networks, peer-group pressure may be lower and diversity of social support higher. Because peers represent different social settings, the ego may benefit from a greater variety of social support, which accumulates and results in greater social capital [51,54,55]. Peers from different settings may therefore serve as social reassurance and buffer the negative consequences of peer rejection [29].

As outlined earlier, the study design does not resolve the degree to which induction and homophily contribute to the strong association between ego’s and alters’ smoking. However, the present data make it easier to study interactions on network structures and dynamics over time, which have implications for the causal directionality of associations: The decreasing influence of age and attenuated peer clustering in smoking in early adulthood suggest that induction (i.e., social contagion of smoking) becomes less important, whereas selection mechanisms endure and continue to affect peer clustering into early adulthood [28–30]. In addition, the results based on *friendship duration* seem to confirm this notion: The ongoing presence of peer influence is supposed to increase peers’ similarity in smoking [56]. The increasing dissimilarity in peer smoking by *friendship duration* as shown in the present study may therefore reflect a selection effect: Smokers rather seek contact with other smokers and reject nonsmokers from their networks. Accordingly, smokers’ tendency to form homogeneous networks may be a consequence of the exclusion of non-smoking peers, implying a dominant deselection mechanism. In long-term friendships that were established before the typical age of smoking initiation, nonsmokers may monitor each other and to greater extent sanction the uptake of smoking. The results presented here are consistent with a recent study that questioned induction as the dominant principle. Instead, the ego-alter homogeneity in smoking was shown to be a result of negative selection: smoking adolescents tend to deselect peers whose smoking behavior becomes dissimilar but retain contact with those who keep smoking [57]. Consequently, long-lasting peer relationships and less dense network formations among smokers may in fact be a consequence of selection processes. It is possible, however, that unobserved confounders contributed to the negative association between *friendship duration* and co-smoking because ego and alter share similar social contexts in which smoking is less common. Likewise, the similarity of egos’ and alters’ behaviors may be caused by joint influences of a third variable [12,58]. Certain social environments with widespread smoking or less restricted access to cigarettes may impose a contextual influence on ego and alter. This notion is not specifically addressed and goes well beyond the focus of the present study, because it would inflict a rather spurious relationship between egos’ and alters’ smoking [58]. Contextual influences are strongly interwoven with induction and homophily, and are consequently difficult to distinguish from the dynamics that arise within peer clusters [54,59].

### Limitations

Some limitations regarding the used data material need to be addressed. Respondents were limited to a maximum of five close friends. If respondents tend to report friends with decreasing “closeness,” and if friendship is driven by homophily, this implies that peer group heterogeneity may be underestimated. Biases and underestimation of smoking due to nonresponse may have occurred, which limits the generalizability of the findings. Sample attrition from the first to the second wave of the survey further reduced the sample size, although additional cases included in the second wave somewhat compensated for this loss. In the balanced sample, the proportion of smokers (S1 Table) and regression coefficients (S2 Table) resemble the results of the unbalanced sample that was examined in this study. Because information on key variables was collected from egos’ ratings, the network variables used may have been miscalculated and peers’ circumstances misjudged. With regard of smoking, for example, egos may over- or underestimate peers’ actual smoking behavior [1]. The two-wave panel survey allowed us to observe smoking patterns during a critical period that includes the transition from late adolescence to early adulthood. However, the two-wave panel design per se is insufficient to determine whether induction or homophily contributed to the presented results. In order to resolve the causal direction of associations, a series of panel waves is required to capture a sufficiently high variation in individuals’ onset and cessation of smoking. Nevertheless, some of the measures used in this study, namely *friendship duration* and *network density*, allowed us to account indirectly for the occurrence of these mechanisms that enhance the ego-alter relationship in smoking.

## Conclusions

This study aims to contribute to our understanding of how social dynamics and structures in peer networks influence smoking. Consistent with prior research, the current study confirms peer clustering in smoking. In adolescence in particular, smoking may function as a catalyst and social lubricant. Substantial gender-differences in the associations between peer clustering in smoking and social network characteristics suggest that young women’s smoking behavior is more socially determined, although the pattern weakens during the transition from late adolescence to early adulthood. The study further proposes that smokers more frequently take advantage of situations to socialize with other smokers. By contrast, nonsmokers’ long-lasting relationships with nonsmoking peers may function as a social corrective and thus contribute to smoking prevention. Although a straightforward account of the role of either homophily or induction is lacking, the results seem to suggest that smokers deselect nonsmokers and nonsmokers deselect smokers from their networks, which consolidates the ego-alter association in smoking behavior.

## Supporting Information

S1 Table Proportion of smokers in subsamples. (DOCX)

S2 Table Random effects logistic regression for the balanced panel: Odds ratios and average marginal effects for daily smoking by gender. (DOCX)
