# Supplementary material for: The Influence of Social Network Characteristics on Peer Clustering in Smoking: A Two-Wave Panel Study of 19- and 23-Year-Old Swedes
Source: PLoS One. 2016 Oct 11;11(10):e0164611. doi: 10.1371/journal.pone.0164611 (PMC5058505; doi:10.1371/journal.pone.0164611)
Supplement: S1 Table — (DOCX) [file pone.0164611.s001.docx]

**S1 Table. Proportion of smokers in subsamples.**

|  | **Respondents in both waves (unbalanced panel) ^(1)^** | |  | **Respondents in both waves (balanced panel)** | |  | **Respondents in either W1 or W2 (independently pooled cross-sections) ^(2)^** | |
| --- | --- | --- | --- | --- | --- | --- | --- | --- |
|  | Wave 1 | Wave 2 |  | Wave 1 | Wave 2 |  | Wave 1 | Wave 2 |
| **Males** |  |  |  |  |  |  |  |  |
| Proportion of smokers | 0.13 | 0.13 |  | 0.12 | 0.13 |  | 0.15 | 0.12 |
| Std.dev. | 0.34 | 0.34 |  | 0.33 | 0.34 |  | 0.35 | 0.33 |
| No. of dyads | 5,726 | 3,056 |  | 2,572 | 2,572 |  | 3,154 | 484 |
|  |  |  |  |  |  |  |  |  |
| **Females** |  |  |  |  |  |  |  |  |
| Proportion of smokers | 0.21 | 0.14 |  | 0.16 | 0.13 |  | 0.25 | 0.18 |
| Std.dev. | 0.41 | 0.34 |  | 0.36 | 0.34 |  | 0.43 | 0.38 |
| No. of dyads | 5,534 | 2,911 |  | 2,440 | 2,440 |  | 3,094 | 471 |

1. Using sample
2. Independently pooled cross-section = unbalanced panel – balanced panel
