# Supplementary material for: The Influence of Social Network Characteristics on Peer Clustering in Smoking: A Two-Wave Panel Study of 19- and 23-Year-Old Swedes
Source: PLoS One. 2016 Oct 11;11(10):e0164611. doi: 10.1371/journal.pone.0164611 (PMC5058505; doi:10.1371/journal.pone.0164611)
Supplement: S2 Table — (DOCX) [file pone.0164611.s002.docx]

**S2 Table. Random effects logistic regression for the balanced panel: Odds ratios and average marginal effects for daily smoking by gender.**

|  | **Males** |  |  |  |  |  | **Females** |  |  |  |  |
| --- | --- | --- | --- | --- | --- | --- | --- | --- | --- | --- | --- |
| **Variables** |  |  |  |  |  |  |  |  |  |  |  |
|  | **OR** | **95% CI** |  | **AME** | **95% CI** |  | **OR** | **95% CI** |  | **AME** | **95% CI** |
|  |  |  |  |  |  |  |  |  |  |  |  |
| Ego: age 23 | **1.56** | (0.88; 2.78) |  | **0.011** | (-0.005; 0.028) |  | **0.89** | (0.53; 1.50) |  | **-0.004** | (-0.025; 0.016) |
|  |  |  |  |  |  |  |  |  |  |  |  |
| Alter smokes | **5.96**** | (4.00; 8.88) |  | **0.045**** | (0.015; 0.076) |  | **6.37**** | (4.46; 9.11) |  | **0.072**** | (0.036; 0.107) |
|  |  |  |  |  |  |  |  |  |  |  |  |
| How often ego/alter meet each other |  |  |  |  |  |  |  |  |  |  |  |
| *Daily* | **Ref.** |  |  | **Ref.** |  |  | **Ref.** |  |  | **Ref.** |  |
| *Several times a week* | **0.82** | (0.52; 1.29) |  | **-0.005** | (-0.019; 0.008) |  | **1.06** | (0.68; 1.68) |  | **0.003** | (-0.018; 0.024) |
| *Once a week* | **0.77** | (0.44; 1.35) |  | **-0.007** | (-0.022; 0.009) |  | **0.60*** | (0.36; 0.99) |  | **-0.019†** | (-0.042; 0.003) |
| *Once a month* | **0.79** | (0.44; 1.43) |  | **-0.006** | (-0.023; 0.010) |  | **0.56*** | (0.33; 0.98) |  | **-0.021†** | (-0.045; 0.002) |
| *Few times a year* | **0.82** | (0.33; 2.00) |  | **-0.006** | (-0.029; 0.018) |  | **0.66** | (0.29; 1.51) |  | **-0.017** | (-0.047; 0.014) |
| *Seldom or never* | **0.65** | (0.17; 2.50) |  | **-0.011** | (-0.040; 0.019) |  | **0.51** | (0.09; 2.75) |  | **-0.024** | (-0.073; 0.024) |
|  |  |  |  |  |  |  |  |  |  |  |  |
| Quality of relationship with alter |  |  |  |  |  |  |  |  |  |  |  |
| *(1) Not good at all* | **Ref.** |  |  | **Ref.** |  |  | **Ref.** |  |  | **Ref.** |  |
| *(2) …* | **3.36** | (0.72; 15.65) | | **0.013†** | (-0.002; 0.028) |  | **1.90** | (0.37; 9.78) |  | **0.017** | (-0.018; 0.053) |
| *(3) …* | **4.64†** | (0.91; 23.68) | | **0.019*** | (0.002; 0.036) |  | **2.02** | (0.37; 10.96) |  | **0.019** | (-0.016; 0.055) |
| *(4) Very good* | **6.49*** | (1.29; 32.73) | | **0.028*** | (0.006; 0.050) |  | **2.41** | (0.42; 14.05) |  | **0.026** | (-0.013; 0.065) |
|  |  |  |  |  |  |  |  |  |  |  |  |
| Trust to alter |  |  |  |  |  |  |  |  |  |  |  |
| *(1) Not at all* | **Ref.** |  |  | **Ref.** |  |  | **Ref.** |  |  | **Ref.** |  |
| *(2) …* | **0.34†** | (0.11; 1.08) |  | **-0.039** | (-0.098; 0.021) |  | **0.86** | (0.34; 2.17) |  | **-0.007** | (-0.051; 0.037) |
| *(3) …* | **0.34†** | (0.11; 1.02) |  | **-0.039** | (-0.098; 0.021) |  | **0.83** | (0.31; 2.26) |  | **-0.008** | (-0.055; 0.039) |
| *(4) Very much* | **0.40** | (0.13; 1.22) |  | **-0.035** | (-0.095; 0.025) |  | **0.71** | (0.24; 2.05) |  | **-0.015** | (-0.065; 0.035) |
|  |  |  |  |  |  |  |  |  |  |  |  |
| Relationship duration |  |  |  |  |  |  |  |  |  |  |  |
| *Between 0 and 19 years* | **0.97†** | (0.93; 1.01) |  | **-0.001** | (-0.002; 0.000) |  | **0.98** | (0.95; 1.01) |  | **-0.001** | (-0.002; 0.000) |
|  |  |  |  |  |  |  |  |  |  |  |  |
| Network density |  |  |  |  |  |  |  |  |  |  |  |
| *Continuous scale from 0 (low density) to* | **2.90†** | (0.93; 8.98) |  | **0.027** | (-0.005; 0.059) |  | **0.97** | (0.36; 2.61) |  | **-0.001** | (-0.040; 0.037) |
| *1 (high density)* |  |  |  |  |  |  |  |  |  |  |  |
|  |  |  |  |  |  |  |  |  |  |  |  |
| Two-way interactions |  |  |  |  |  |  |  |  |  |  |  |
| *Alter smokes × Age* | **0.85** | (0.47; 1.54) |  |  |  |  | **0.38**** | (0.20; 0.70) |  |  |  |
| *Alter smokes × How often ego/alter meet* | **0.78** | (0.58; 1.05) |  |  |  |  | **0.83** | (0.64; 1.08) |  |  |  |
| *Alter smokes × Quality of relationship* | **1.26** | (0.80; 2.00) |  |  |  |  | **1.57*** | (1.02; 2.42) |  |  |  |
| *Alter smokes × Trust to alter* | **1.04** | (0.74; 1.46) |  |  |  |  | **1.41†** | (0.97; 2.05) |  |  |  |
| *Alter smokes × Relationship duration* | **0.99** | (0.94; 1.05) |  |  |  |  | **0.99** | (0.95; 1.05) |  |  |  |
| *Alter smokes × Network density* | **0.70** | (0.21; 2.35) |  |  |  |  | **8.34**** | (2.42; 28.68) |  |  |  |
|  |  |  |  |  |  |  |  |  |  |  |  |
| Three-way interactions |  |  |  |  |  |  |  |  |  |  |  |
| *Alter smokes × Age × How often ego/alter meet* | **0.65** | (0.39; 1.09) |  |  |  |  | **0.99** | (0.65; 1.52) |  |  |  |
| *Alter smokes × Age × Quality of relationship* | **0.55** | (0.24; 1.27) |  |  |  |  | **0.96** | (0.42; 2.21) |  |  |  |
| *Alter smokes × Age × Trust to alter* | **1.07** | (0.55; 2.09) |  |  |  |  | **1.01** | (0.50; 2.04) |  |  |  |
| *Alter smokes × Age × Relationship duration* | **1.06** | (0.96; 1.17) |  |  |  |  | **1.00** | (0.90; 1.11) |  |  |  |
| *Alter smokes × Age × Network density* | **0.41** | (0.03; 5.81) |  |  |  |  | **0.87** | (0.06; 12.52) |  |  |  |
|  |  |  |  |  |  |  |  |  |  |  |  |
| No. of dyads | 5,144 |  |  |  |  |  | 4,880 |  |  |  |  |
| No. of individuals | 685 |  |  |  |  |  | 643 |  |  |  |  |

† *p* < 0.10; * *p* < 0.05; ** *p* < 0.01

All variables mutually adjusted, including migration background, employment status, civil status and educational attainment (coefficients not shown)

Random effects parameters not shown
